# In-Band RCS Reduction of Circularly Polarized Antenna Array with High Aperture Efficiency and Low Profile for Satellite and Navigation Systems

**DOI:** 10.3390/mi17101106

**Published:** 2026-09-22

**Authors:** Gang Shi, Zhenzhen Yue, Siwen Wang, Yongtao Jia, Jiahao Zhang, Xiao Tao, Zhong-Xun Liu, Haoyu Lei, Ying Liu

**Affiliations:** 1 National Key Laboratory of Radar Detection and Sensing, Xidian University, Xi’an 710071, China; kobeport@163.com (G.S.); 18829033095@163.com (S.W.); jiazong0629@163.com (Y.J.); liuzhongxun@xidian.edu.cn (Z.-X.L.); leihaoyu@xidian.edu.cn (H.L.); liuying@mail.xidian.edu.cn (Y.L.); 2 Xi’an Institute of Space Radio Technology, Xi’an 710100, China; yuezhenzhen@163.com (Z.Y.); smilecast504@163.com (X.T.)

**Keywords:** antenna array, circularly polarized, characteristic mode analysis, high aperture efficiency, low profile, radar cross section reduction

## Abstract

This paper proposes a circularly polarized (CP) antenna array featuring high aperture efficiency, a low in-band radar cross section (RCS), and a low profile. First, two different patch antenna elements are designed, which exhibit desirable CP performance and a 180° reflection phase difference under x-polarized normal incidence within the same operation band. These elements are then arranged in a chessboard configuration to simultaneously achieve high aperture efficiency and in-band RCS reduction under x-polarized normal incidence. Subsequently, the parasitic units are uniformly embedded into the array elements, which are employed to realize reflection phase cancellation with the array elements under y-polarization normal incidence. The simulated results show that the proposed array achieves a peak monostatic RCS reduction of 12.8 dB, and the 6 dB RCS reduction bandwidth fully covers the antenna’s operating bandwidth under both x- and y-polarized normal incidences. Moreover, the gain of the antenna array reaches 18.2 dBic at 3 GHz with an aperture efficiency of 58% and a profile of 0.03λ. A good agreement is obtained between the simulated and measured results. This design successfully combines the advantages of high aperture efficiency, dual-polarized in-band RCS reduction, and a low profile, making it highly valuable for aerospace satellite communications and unmanned aerial vehicle data links.

## 1. Introduction

With the rapid deployment of low earth orbit (LEO) satellite constellations and unmanned aerial vehicles (UAV), high-performance circularly polarized (CP) antenna arrays have become indispensable for modern air and space systems due to their anti-interference capability. However, micro-aerospace platforms such as CubeSats and stealth drones operate under stringent size and weight constraints. These platforms demand CP antennas that not only exhibit high aperture efficiency to maintain reliable communications but also feature a low radar cross section (RCS) to prevent detection. Achieving high aperture efficiency inherently requires a large effective radiating aperture, which inevitably increases structural scattering. Consequently, resolving the physical trade-off among high aperture efficiency, low-observable capabilities, and ultra-low-profile miniaturization remains a critical challenge in aerospace systems.

Absorbing radar energy is the most effective method to realize RCS reduction by using absorbing materials or resistors. However, this approach will inevitably affect antenna performance, which is usually limited to the antenna out-of-band RCS reduction [1]. Due to their flexible electromagnetic functions and low profile, metasurfaces have been extensively applied to reduce CP antenna array RCS through a phase cancellation mechanism [2]. The low RCS property is realized by reflection phase cancellation from two adjacent metasurfaces in the chessboard configuration [3,4]. These chessboard metasurface structures are then utilized to reduce CP antenna array RCS. A chessboard configuration is typically constructed using polarization conversion metasurfaces (PCM), which are loaded either above or below the CP antenna array. Although the wideband RCS reduction and gain enhancement can be achieved simultaneously, combining an antenna array with separate metasurfaces inevitably increases the overall profile and structural volume [5,6,7]. To satisfy stringent miniaturization requirements, metasurface-inspired antennas serve as promising candidates due to their low profiles. Well designed artificial magnetic conductors (AMC) can directly serve as antenna-radiating elements. Backward scattering is reduced due to reflected phase cancellation between the AMCs and the ground plane [8]. Similar to this method, PCM is used as a radiator, and three kinds of AMC units are arranged around the PCM. In this way, wideband RCS reduction is passively achieved [9]. To further increase the RCS reduction bandwidth, PCMs based on the chessboard configuration are excited by the sequentially rotated feeding networks [10,11,12,13,14,15]. However, it is preferable to use CP elements rather than linearly polarized (LP) elements when designing high-gain CP antenna arrays. Conventional PCM units typically generate LP waves, leading to a decline in the aperture efficiency of the antenna array.

In this paper, a low-profile CP antenna array featuring high aperture efficiency and low in-band RCS is proposed, as shown in Figure 1. The CP radiation performance of two kinds of patch elements is investigated by characteristic mode analysis. Two patch elements are arranged in the chessboard configuration to obtain high aperture efficiency. Low in-band RCS property is realized by the interference cancellation of reflected waves between patch elements and parasitic units under x- and y-polarized normal incidences. The rest of this paper is organized as follows: In Section 2, the proposed CP antenna array is presented. The simulated and measured results are discussed in Section 3. Finally, a brief conclusion is drawn in Section 4.

## 2. Antenna Design

The geometry of two kinds of CP patch elements is presented in Figure 2. The two CP patch elements are composed of three layers: the bottom layer is the metallic ground; the middle layer is the substrate with εr = 2.1; and the top layer is the patch. The patch of element 1 has a pair of truncated corners to generate CP performance. In addition to the truncated corners, two pairs of orthogonal strips are symmetrically connected to the patch of element 2. Moreover, four shorting pins and two 1000 Ω resistors are inserted symmetrically in the vertical strip.

The resonant frequency and radiation performance of the two patch elements are analyzed using characteristic mode theory. As discussed in [16], CP performance is achieved when a pair of orthogonal modes with equal modal significance (MS) and a 90° characteristic angle (CA) is excited simultaneously. The characteristic mode analysis of both patch elements is performed by FEKO. All other simulations were performed by HFSS. As shown in Figure 3, modes 1 and 2 of patch element 1 resonated at 2.97 GHz and 3.05 GHz, respectively. The MS curves of modes 1 and 2 intersect, and their CA difference is close to 90° at 3.01 GHz. Furthermore, the current distributions of modes 1 and 2 are orthogonal at 3.01 GHz. They both generate broadside radiation patterns. Therefore, modes 1 and 2 of patch element 1 can be selected as the operation modes to generate CP performance.

The modal properties of patch element 2 are shown in Figure 4. The MS curves of modes 2 and 4 exhibit equal magnitude, while their CA difference approaches 90° at 2.98 GHz. It can be seen that the currents of the two modes are mainly concentrated in the central patch at this frequency. The currents distribution of the two modes is similar but in the orthogonal directions. Moreover, the two modes both generate broadside radiation patterns. Thus, patch element 2 can generate CP performance when modes 2 and 4 are properly excited.

To generate desirable CP performance, the two patch elements are excited by the probes where the current distribution of orthogonal modes are strong. They both have a good axial ratio (AR) performance at 3 GHz, as shown in Figure 5. The radiation patterns of both patch elements exhibit similar LHCP performance at 3 GHz. Due to the introduced resistors, the gain of patch element 2 is 0.4 dB lower than that of patch element 1.

The reflected properties of two patch elements are simulated by the periodic boundary conditions and floquet excitations. Since two patch elements have similar CP performance in the same operation band, those reflected magnitudes are close in Figure 6a. Under these conditions, the reflection phase difference between them becomes the critical factor for low-RCS design. The reflected phase of patch element 1 and patch element 2 under x-polarized normal incidence is shown in Figure 6b. It can be seen that the reflected phase difference of two patch elements can reach 180° ± 37° in the operation band. This phenomenon is attributed to the orthogonal strips and high-value resistors introduced in patch element 2.

The electrical size of patch element 2 in the x-direction is significantly increased. The current path on patch element 2 is longer than that of patch element 1 at 3 GHz under x-polarized normal incidence, as shown in Figure 7. The current distributions on the strips are significantly affected by the introduced high-value resistors without compromising radiation performance. As shown in Figure 8a, compared with patch element 2 without resistors, the reflection phase of patch element 2 with resistors exhibits an abrupt change within the operating band. The variation in the reflection phase of the frequency band shifts toward a lower frequency with the increase of *L*_2_. Thus, the reflection phase of patch element 2 can be flexibly tuned by adjusting the position of the resistors. To provide deeper physical insight into this mechanism, a simplified equivalent circuit model is introduced and integrated, as shown in Figure 8b. The vertical strip loaded with the resistor and shorting pin functions as a short-circuited transmission-line stub. Shifting the resistor position *L*_2_ alters the effective electrical length of this stub. Specifically, an increase in *L*_2_ enlarges the equivalent series inductance of the input impedance. This added inductive reactance shifts the resonant zero-crossing point of the reflection phase toward lower frequencies. Crucially, since the CP radiation currents concentrate at the patch center, tuning the peripheral reactance via *L*_2_ adjusts the scattering phase without degrading radiation efficiency, as shown in Figure 8c.

As discussed in [3], a 10 dB RCS reduction can be realized based on the chessboard configuration composed of patch element 1 and patch element 2 under x-polarized normal incidence. However, the physical geometry of the two elements along the y-axis are almost the same, leading to a small, reflected phase difference. In order to further achieve a 10 dB RCS reduction under y-polarized normal incidence, a parasitic unit is designed delicately to provide phase cancellation against both patch elements. As shown in Figure 9, the geometry of the parasitic unit is the same as that of two patch elements, while the patch of the parasitic unit has a pair of truncated corners in the opposite diagonal and four strips. It can be seen that the parasitic unit maintains an acceptable reflection magnitude difference and an approximate 180° phase difference relative to both patch elements in the operation band under y-polarized normal incidence, as shown in Figure 10.

A pairwise controlled sensitivity study was carried out at 3 GHz, as summarized in Table 1. Under y-polarized normal incidence, the incident electric field aligns with the y-axis, causing the induced surface currents on the parasitic element to flow predominantly along the y-direction. Consequently, the structural parameters associated with the y-axis directly have an evident impact on the reflected phase. In contrast, the dimensions oriented along the x-axis exhibit negligible influence due to polarization orthogonality.

Based on the chessboard configuration composed of two patch elements, parasitic units are integrated uniformly among the array elements to reduce the monostatic in-band RCS of the antenna array without increasing the overall size when the normal incidence is y-polarized. To minimize mutual coupling between parasitic units and array elements, the array elements spacing is set to 0.75 λ, as shown in Figure 11.

For ease of illustration, the numbers marked on the patch denote the port names. Here, the coupling coefficients of the four middle elements have been analyzed because they are fully surrounded by neighboring radiators and parasitic units. It can be observed that all mutual coupling levels remain strictly below −24 dB in the operation band with or without the parasitic units. The parasitic units do not induce the degradation of coupling between array elements, as shown in Figure 12. Moreover, the radiation patterns of the antenna array are basically similar in these two cases.

The array performance is comparatively investigated with and without parasitic loading, as depicted in Figure 13. Under the radiation case, because the parasitic elements are passive and deliberately embedded among the array elements, the reflection coefficient and gain of the proposed antenna array without or with parasitic units remain almost similar. Under the scattering case, because the parasitic elements are arranged symmetrically within the lattice, their secondary scattering does not disrupt the primary phase cancellation between the chessboard elements. Therefore, the x-polarized normal monostatic RCS of the proposed antenna array without or with parasitic units remain almost similar.

The chessboard surface is excited directly by a power divider whose outputs have the same equal magnitude and phase, exhibiting the high aperture efficiency. Moreover, several key parameters of the proposed antenna array are optimized to simultaneously achieve the satisfactory radiation and scattering performance in the operation band.

## 3. Simulated and Measured Results

The 4 × 4 CP antenna array is fabricated to verify the design method, as shown in Figure 14. The simulated and measured radiation performance of the proposed antenna array is shown in Figure 15. The simulated impedance bandwidth and 3dB AR bandwidth range between 3 and 3.03 GHz. Although the measured impedance bandwidth and 3dB AR bandwidth of the proposed array shifts toward a higher frequency, the simulated and measured curves have a similar tendency. The slight discrepancies are attributed to fabrication tolerances and measurement errors. In addition, the simulated boresight gain of the proposed antenna array is 18.2 dBic at 3 GHz with an aperture efficiency of 58%. To further quantify internal dissipations, the simulated and measured radiation efficiencies of the array are reported as 77.1% and 72.5% at 3 GHz, respectively. The loss budget of the proposed array mainly accounts for dielectric substrate losses, power absorption by the resistors, and insertion losses of the power divider. The measured result has a good agreement with the simulated one.

To verify the scattering performance of the proposed antenna array, a conventional 4 × 4 square array of element 1 is chosen as the reference antenna. Moreover, both antenna arrays have the same size and are terminated with matched loads during scattering measurements. The simulated and measured monostatic RCS of the reference array and proposed array are shown in Figure 16. Compared with the reference array, the simulated monostatic RCS of the proposed array is significantly reduced across the operating band under normal incidence for both polarizations. Moreover, the out-of-band RCS of the proposed array is reduced simultaneously due to the introduced resistors under x-polarized normal incidence. A good agreement is obtained between the simulated and measured results of the proposed antenna.

The 3-D bistatic RCS of the reference array and the proposed array at 3 GHz are given in Figure 17, respectively. Due to the phase cancellation, the scattering power of the proposed array is deflected into four directions. Since the four redirected scattering lobes are symmetrically aligned along the diagonal planes of the chessboard configuration, the 2-D bistatic RCS pattern along the diagonal cut (phi = 45°) under y-polarized normal incidence at 3 GHz is shown in Figure 17c. Along the boresight direction, the proposed array attains a remarkable null depth with an RCS reduction of 12.8 dB relative to the reference array. As demonstrated along the diagonal phi = 45° cut, destructive interference redirects the specular reflection away from the normal axis into four diagonal beams peaking at theta = ±25° with a maximum level of −5.5 dBsm.

To further demonstrate the advantages of our design, Table 2 presents the comparison between the proposed design and the reported low RCS CP antenna arrays. It can be seen that the prior works have advantages in BW&AR bandwidth and RCS reduction bandwidth, while this work excels in aperture efficiency and profile. Most prior works compare RCS reduction relative to a metal plate, which naturally yields broad bandwidths and high peaks due to strong specular reflection. In contrast, our RCS reference is chosen as an identically sized, matched CP reference antenna array. Because a matched antenna already exhibits much lower baseline scattering than a metal plate, achieving over 10 dB in-band reduction against an actual antenna is physically far more challenging and meaningful.

The proposed antenna array exhibits a relatively narrow operating bandwidth of approximately 1.5%. It should be emphasized that simultaneously satisfying the conditions for characteristic mode orthogonality (for CP radiation) and the 180° reflection phase cancellation (for dual-polarized RCS reduction) within a low profile of 0.03 $\lambda_{0}$ inherently limits the operational bandwidth. Furthermore, the achieved operating bandwidth is highly suitable for satellite communications and UAV data links, where high aperture efficiency and low observability are strictly prioritized over wideband operation.

## 4. Conclusions

A CP antenna array with high aperture efficiency and low in-band RCS based on the co-design of radiation and scattering is proposed in this paper. This co-design strategy transforms the CP patch elements directly into a functional phase-cancellation surface. The proposed CP antenna array simultaneously achieves high aperture efficiency, low in-band RCS, and a low profile, making it highly valuable for aerospace satellite communications and unmanned aerial vehicle data links.

## Figures and Tables

**Figure 1:**
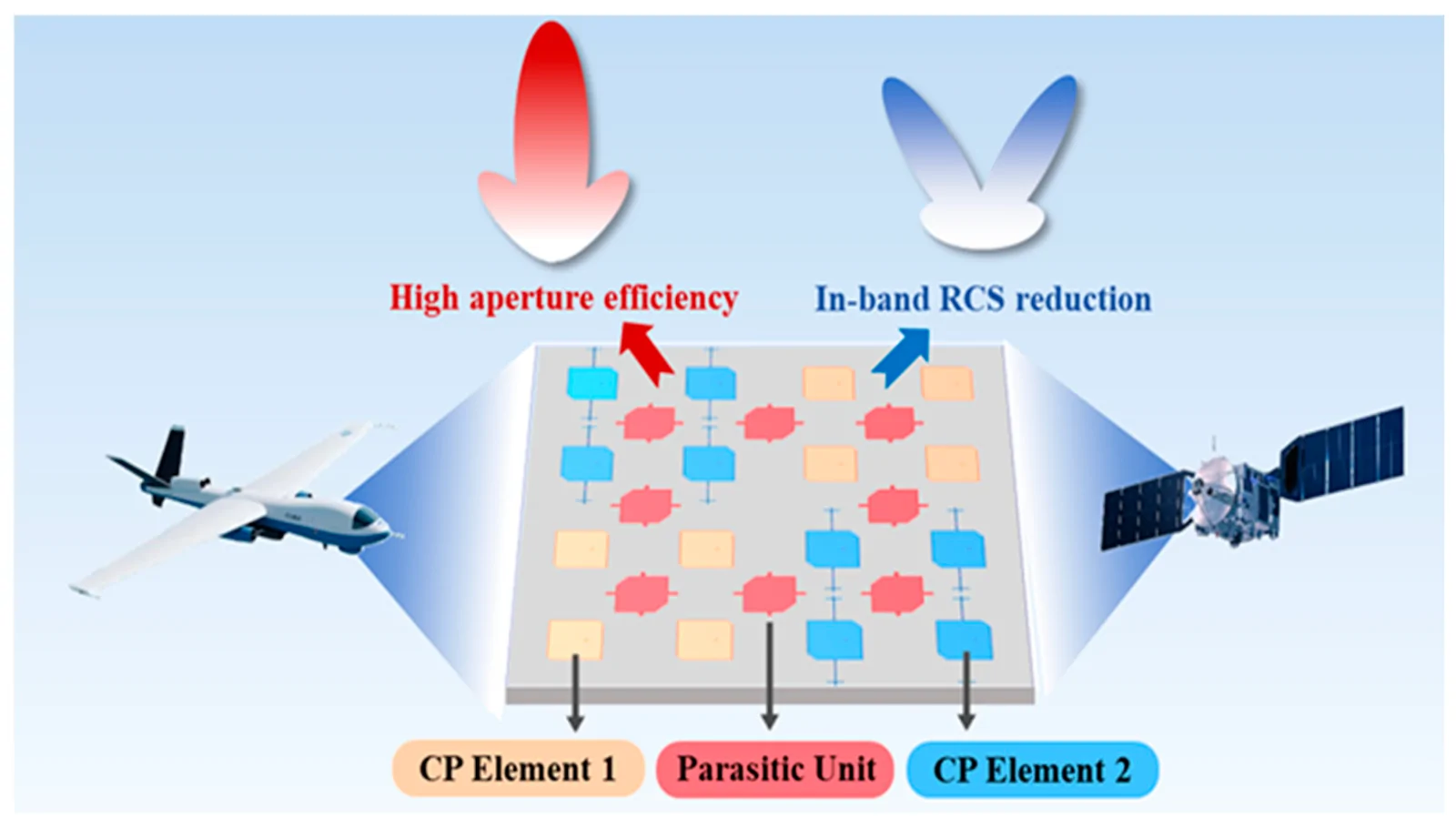
Conceptual illustration of the proposed antenna application scenario.

**Figure 2:**
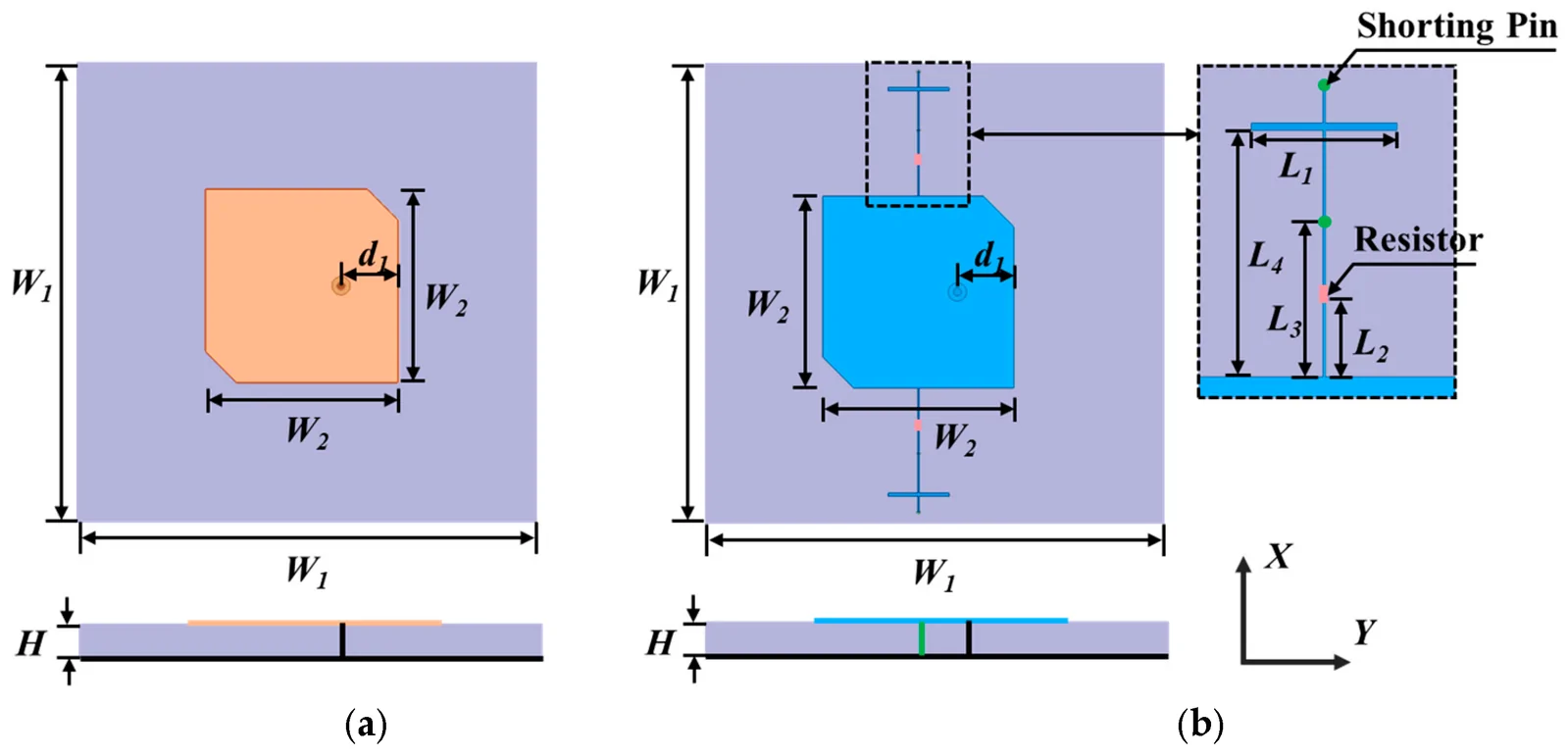
The geometry of two kinds of CP patch elements (**a**) element 1. (**b**) element 2. All dimensions: *W*_1_ = 75, *W*_2_ = 32.9, *d*_1_ = 6.4, *L*_1_ = 10, *L*_2_ = 2.55, *L*_3_ = 13.05, *L*_4_ = 16.55, *H* = 1.75. Unit: mm.

**Figure 3:**
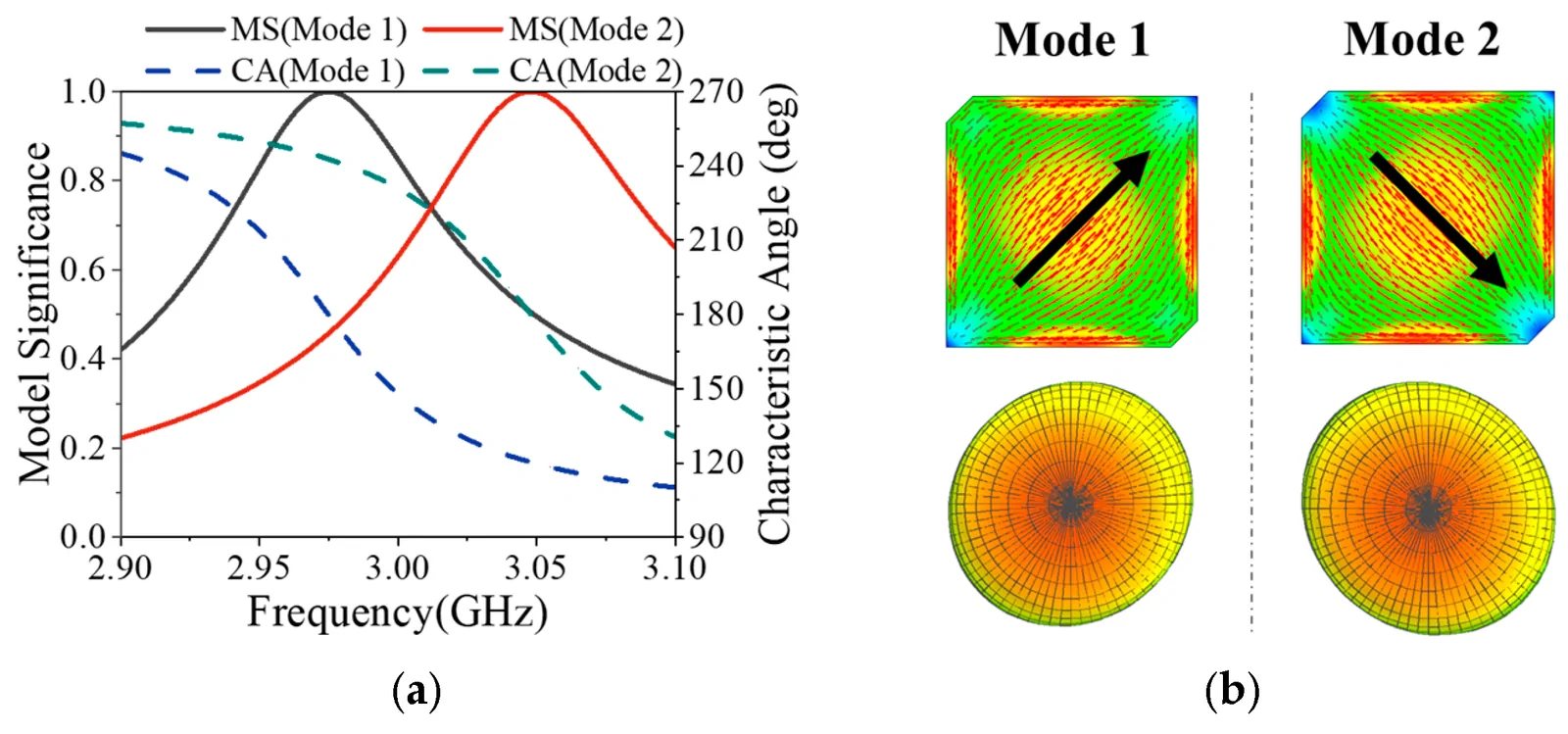
The modal properties of patch element 1 (**a**) MS and CA. (**b**) Currents and Radiation patterns at 3.01 GHz.

**Figure 4:**
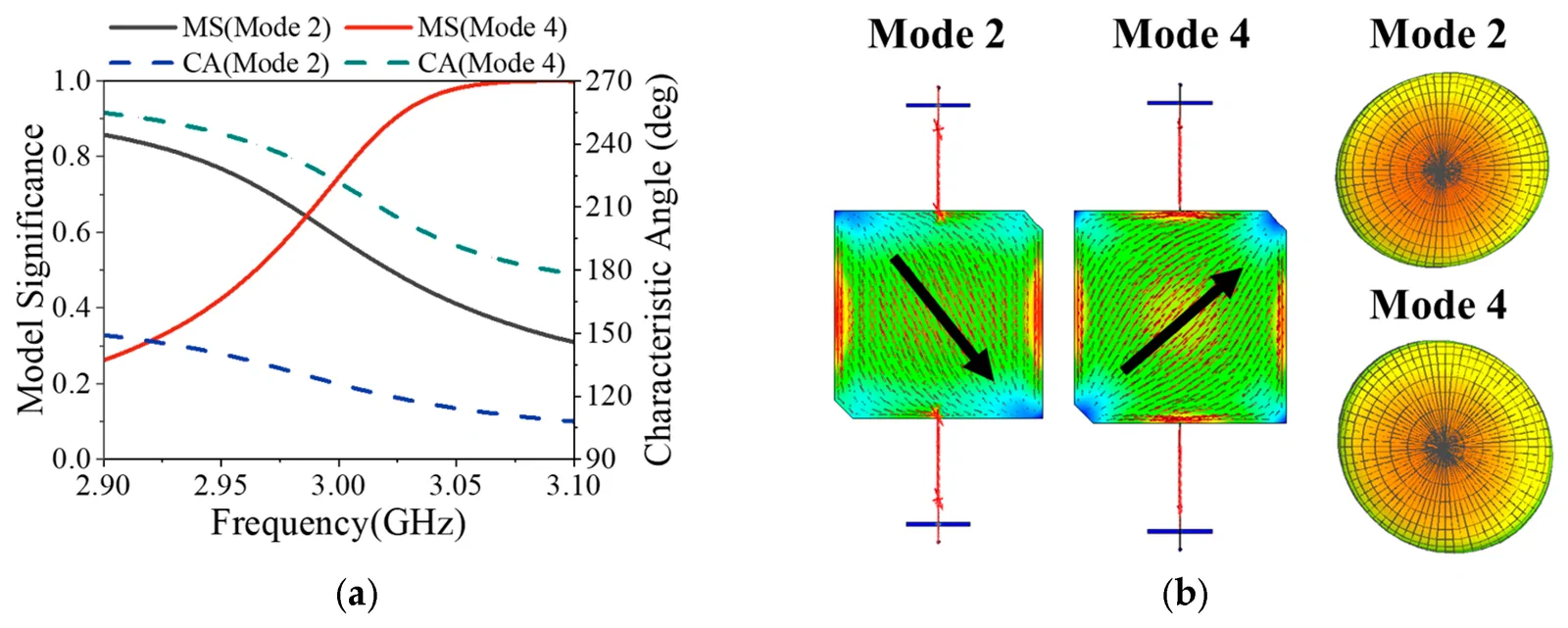
The modal properties of patch element 2 (**a**) MS and CA. (**b**) Currents and Radiation patterns at 2.98 GHz.

**Figure 5:**
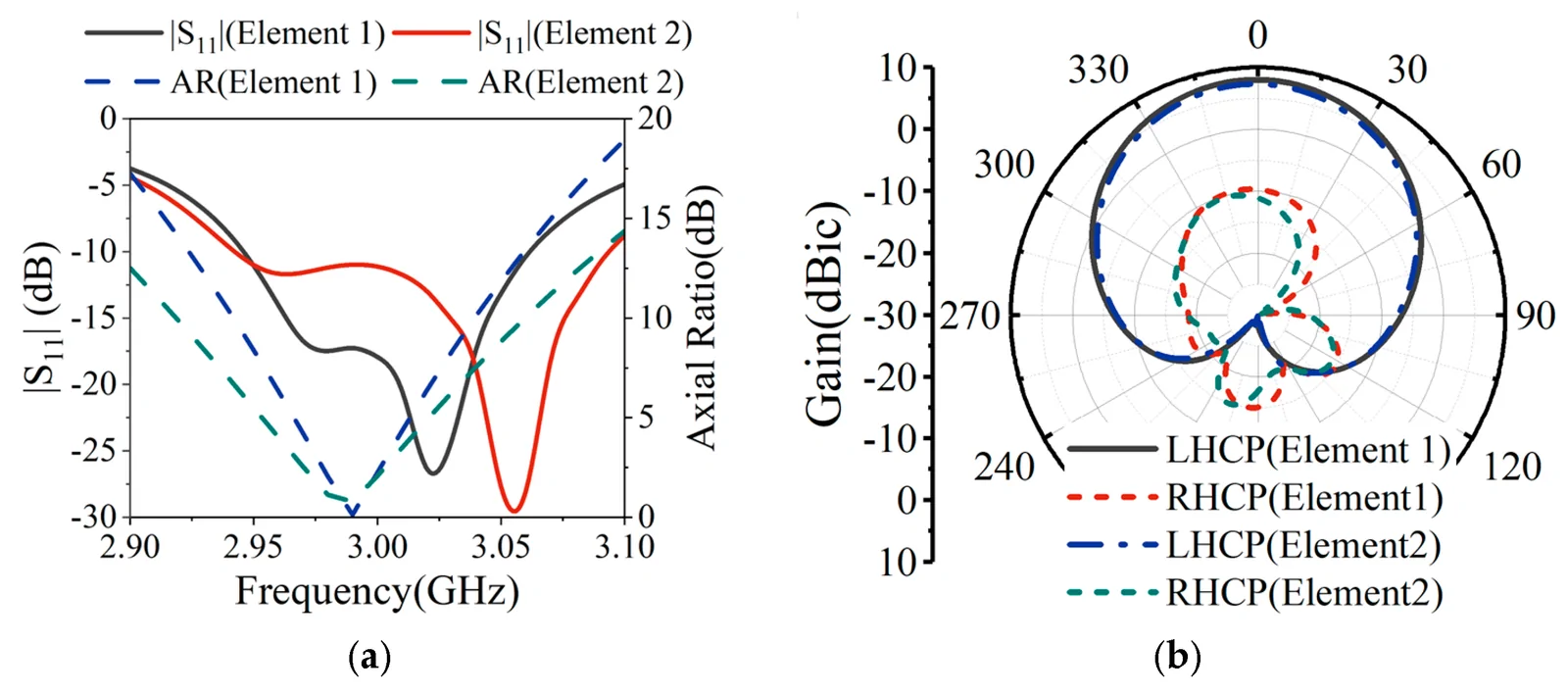
The performance of two patch elements (**a**) *S*-parameters and AR. (**b**) Radiation patterns at 3 GHz.

**Figure 6:**
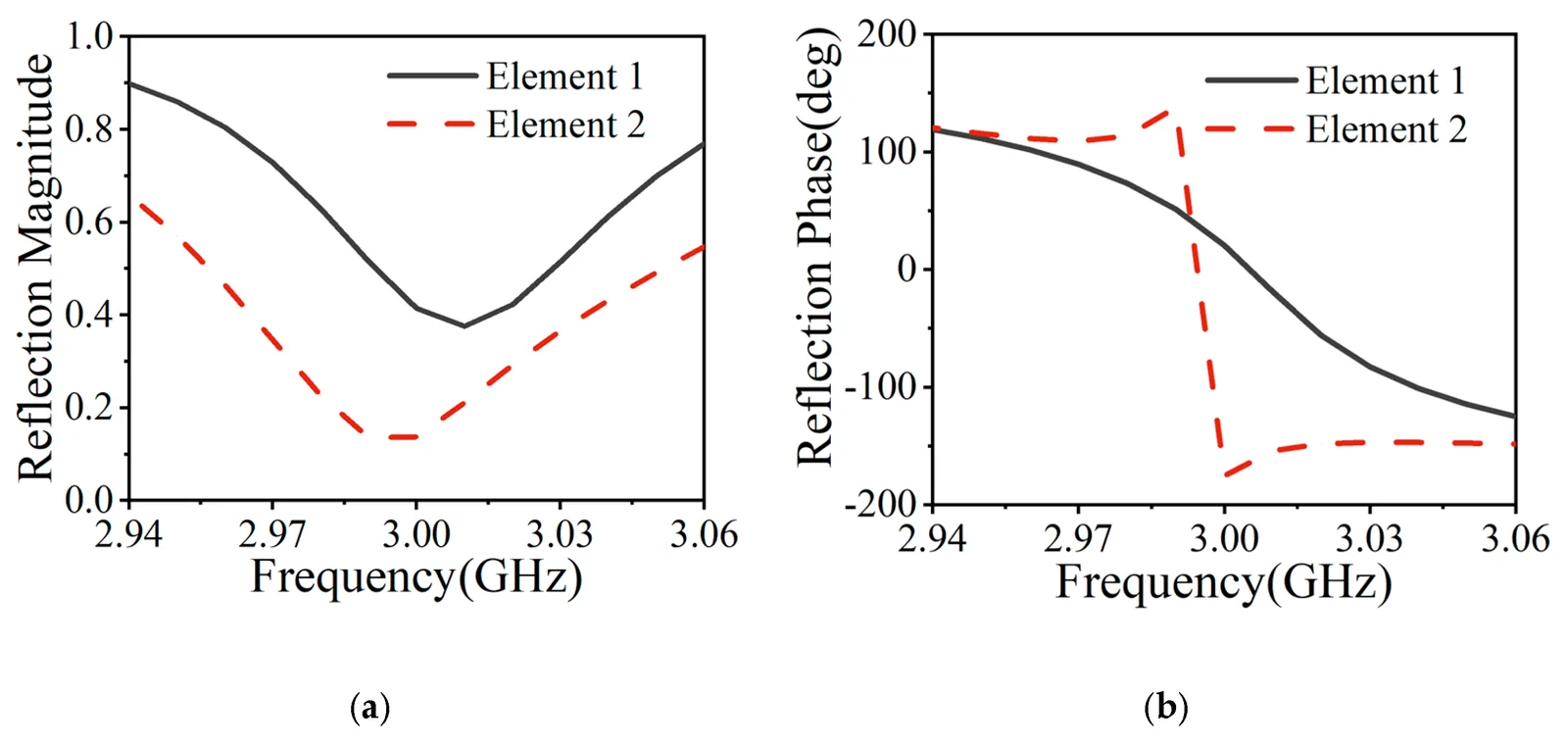
Two patch elements under x-polarized normal incidence (**a**) Reflected magnitudes. (**b**) Reflected phases.

**Figure 7:**
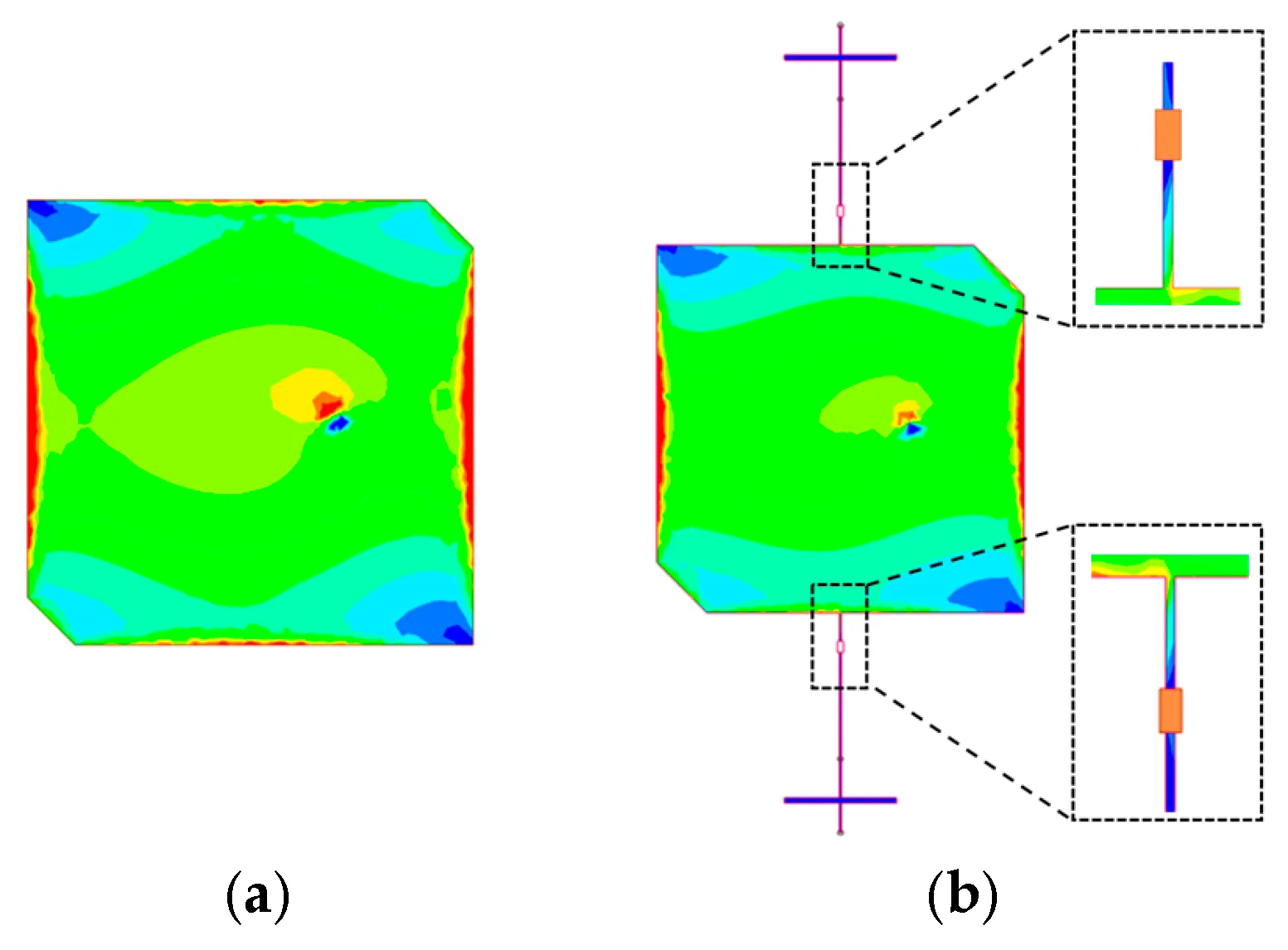
Simulated currents on the two patch elements at 3 GHz when the normal incidence is x-polarized (**a**) patch element 1. (**b**) patch element 2.

**Figure 8:**
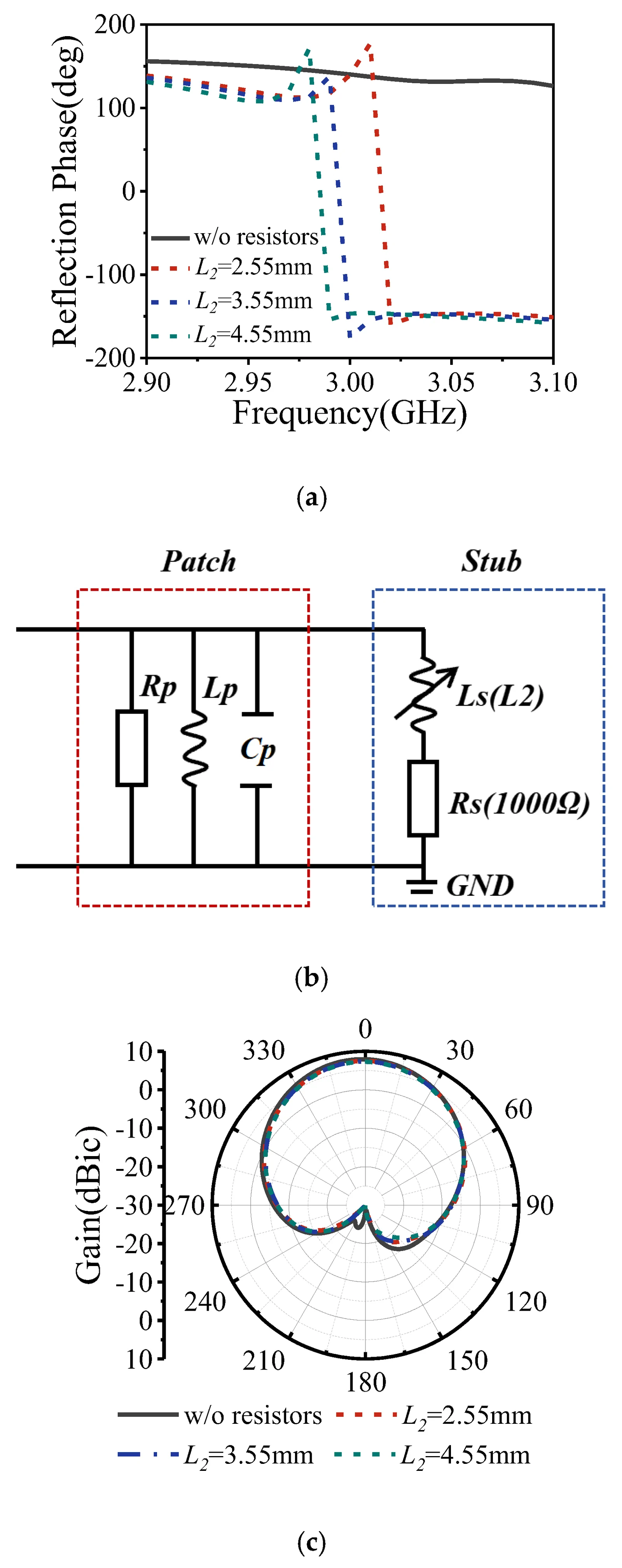
Patch element 2 without resistors and loaded resistors with different *L*_2_: (**a**) Reflected phases under *x*-polarized normal incidence. (**b**) Equivalent circuit model. (**c**) Radiation patterns at 3 GHz.

**Figure 9:**
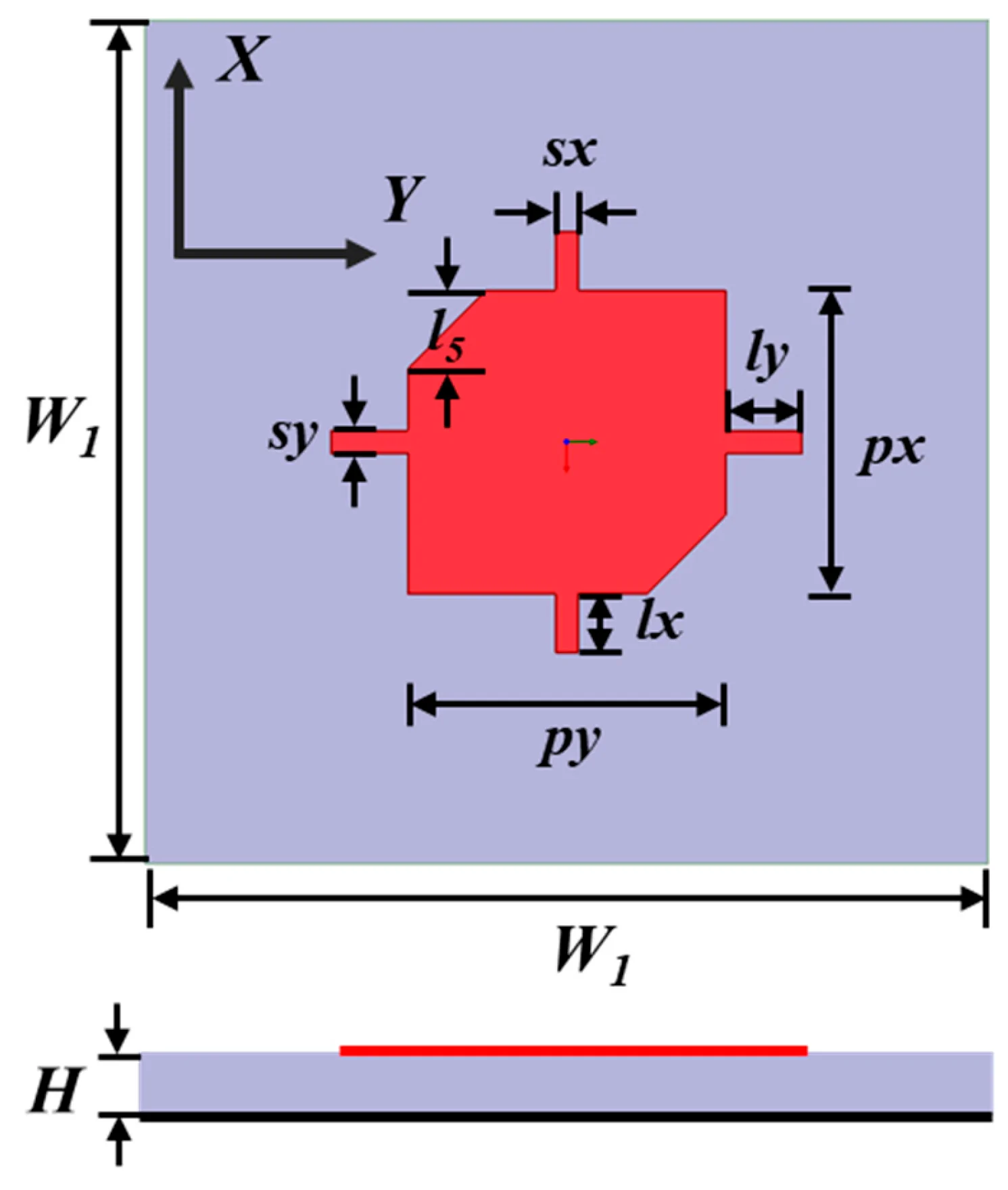
The geometry of the parasitic unit. All dimensions: *W*_1_ = 75, *px* = 30.5, *py* = 31.5, *sx* = 2, *sy* = 2, *lx* = 2.75, *ly* = 3.55, *L*_5_ = 7, *H* = 1.75. Unit: mm.

**Figure 10:**
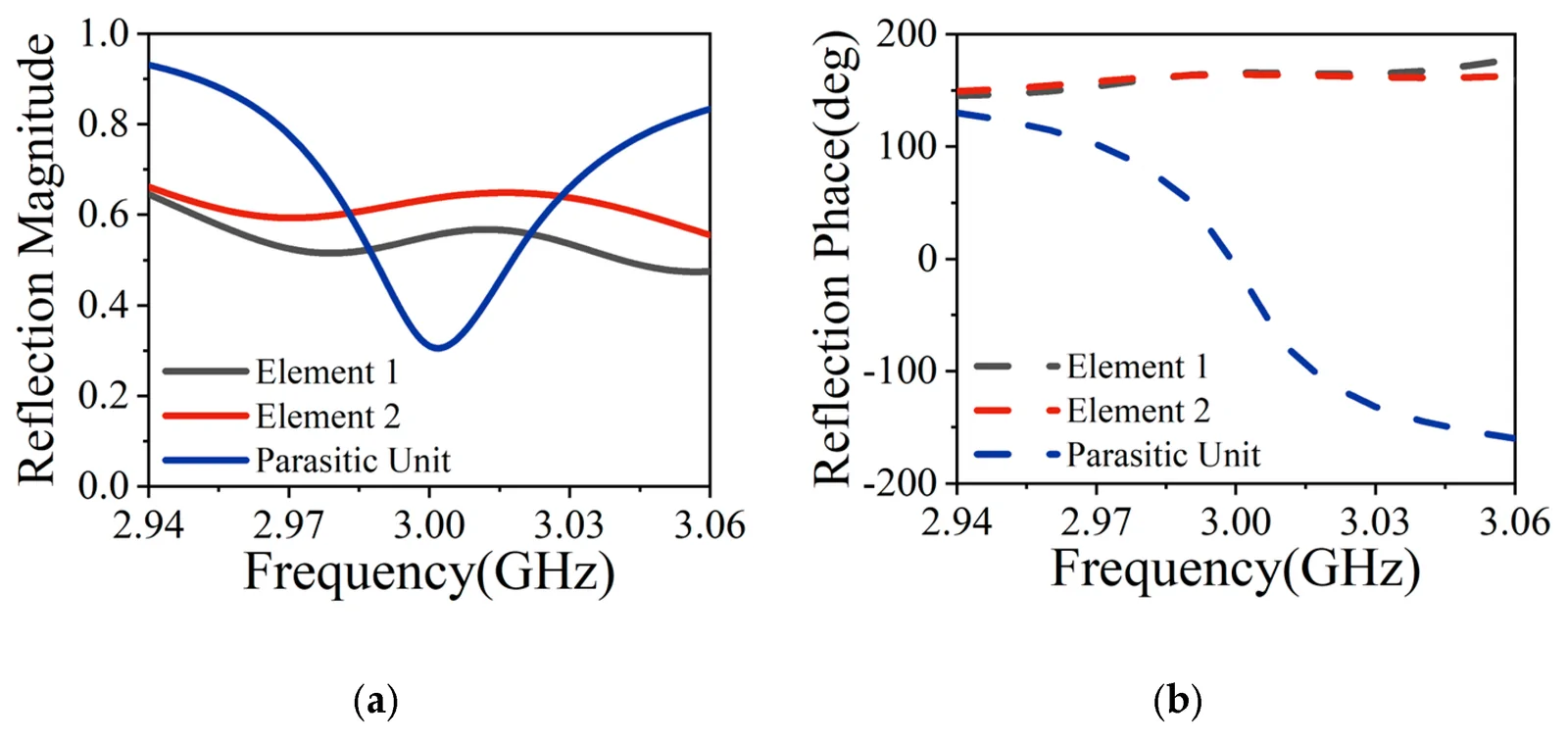
Two patch elements and parasitic unit under y-polarized normal incidence (**a**) Reflected magnitudes. (**b**) Reflected phases.

**Figure 11:**
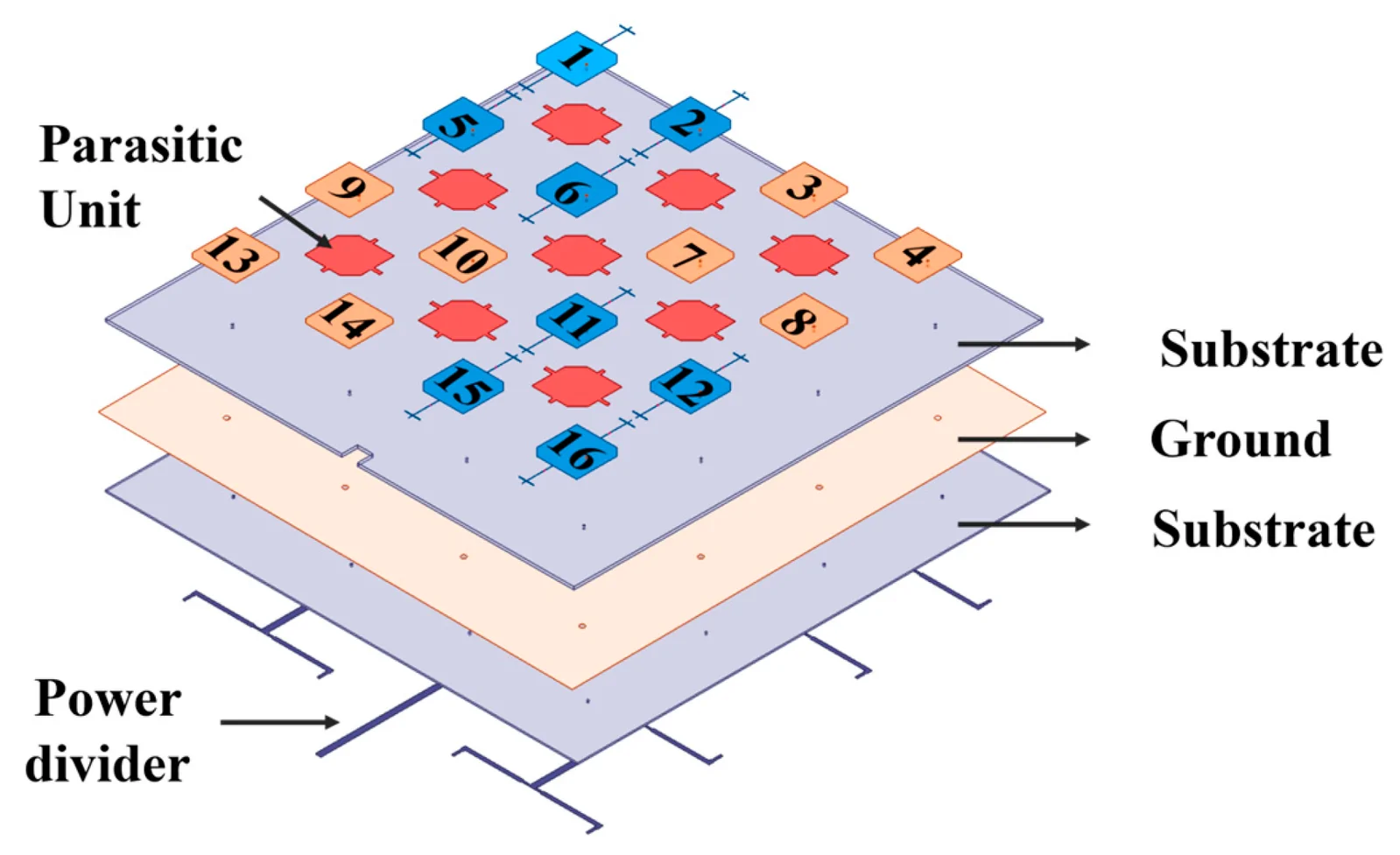
The schematic view of the proposed antenna array. Optimized parameters: *L*_5_ = 5.3, *px* = 29, *py* = 29, *lx* = 3.5, *ly* = 5.85. Unit: mm.

**Figure 12:**
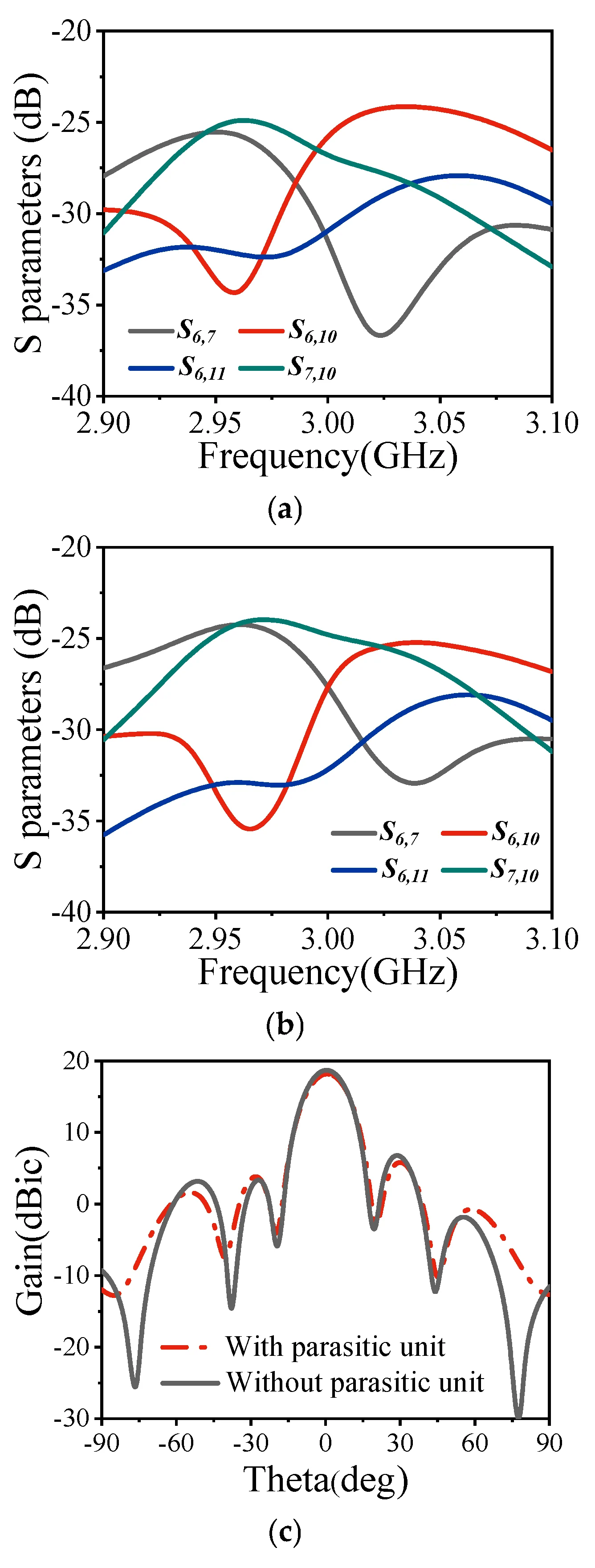
The proposed antenna array: (**a**) Coupling coefficients without the parasitic units. (**b**) Coupling coefficients with the parasitic units. (**c**) The radiation patterns array at 3 GHz.

**Figure 13:**
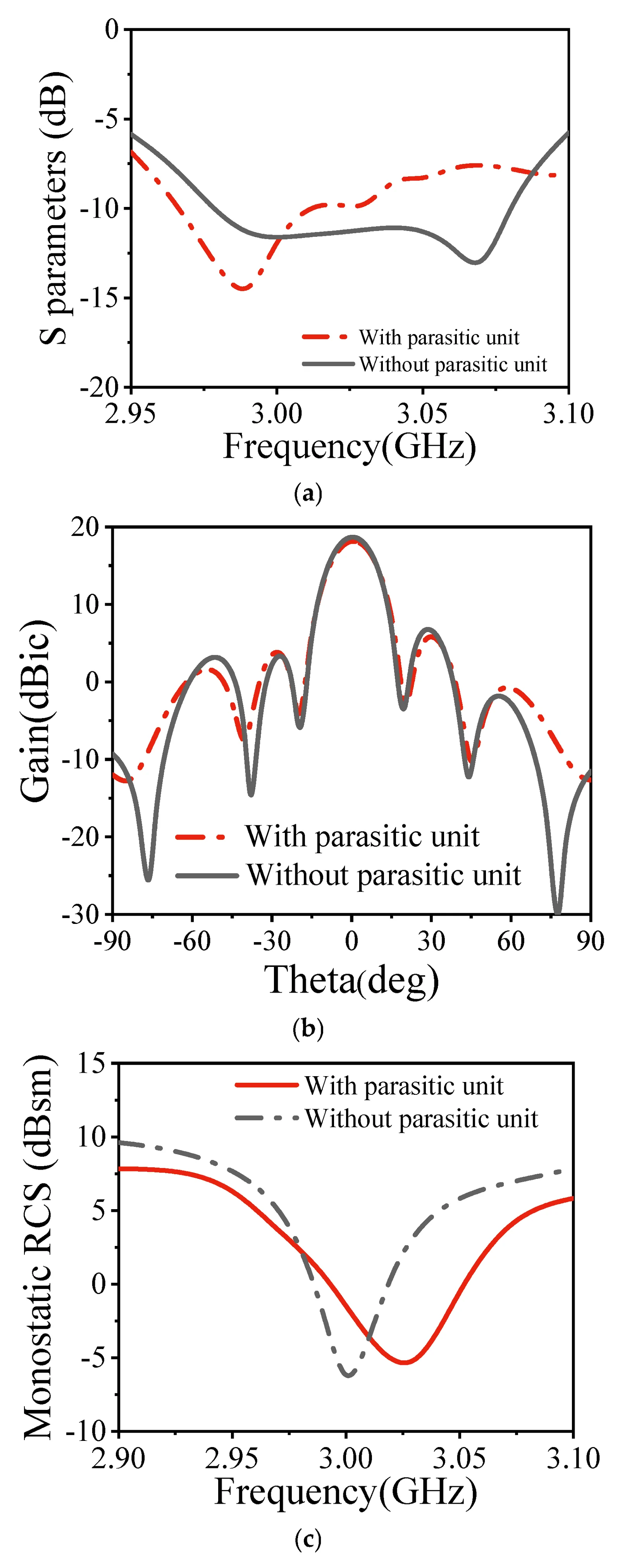
The proposed antenna array without or with parasitic units: (**a**) Reflection coefficient. (**b**) Gain. (**c**) x-polarized normal monostatic RCS.

**Figure 14:**
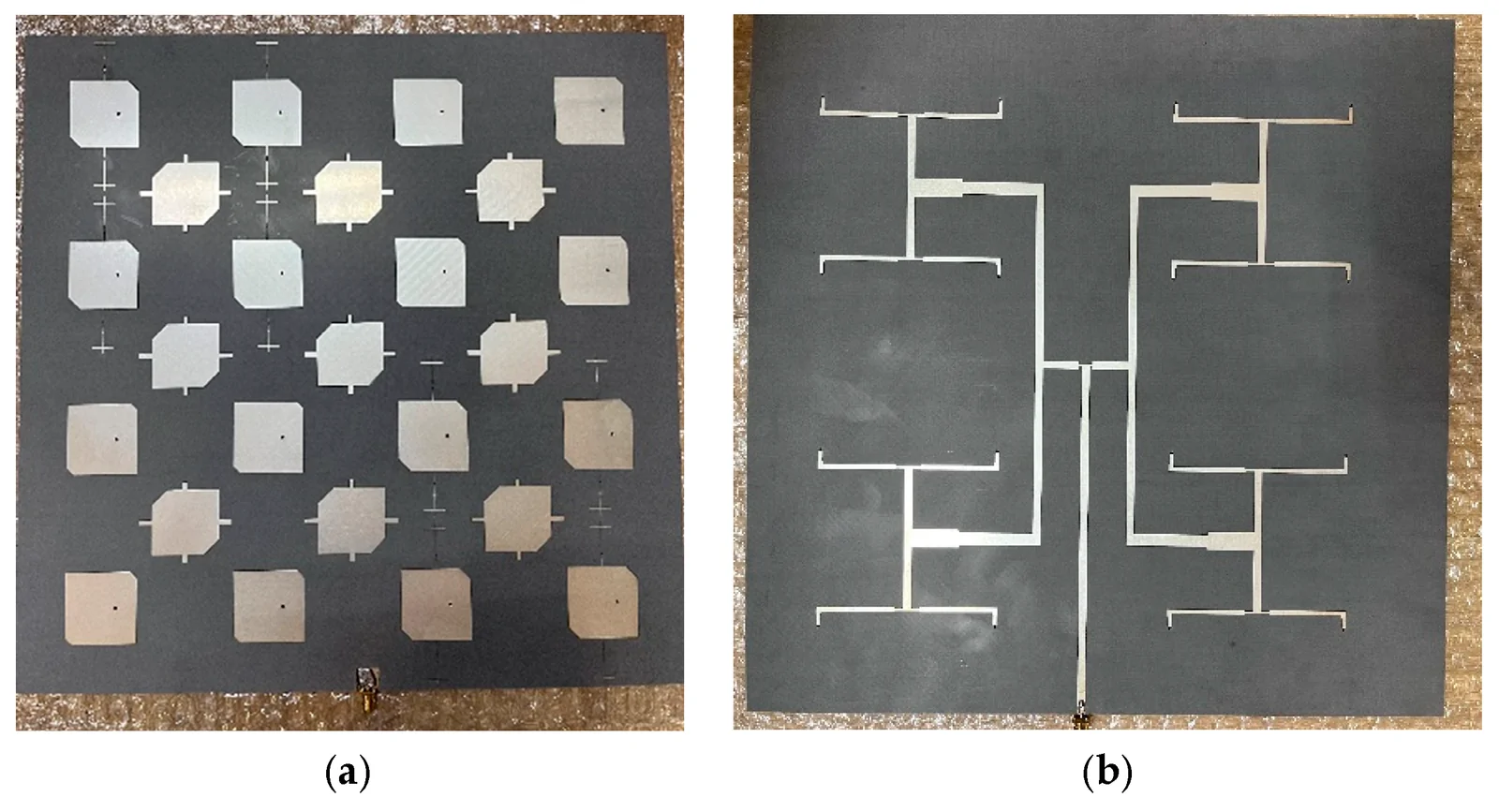
Prototypes of the proposed array (**a**) Top view. (**b**) Bottom view.

**Figure 15:**
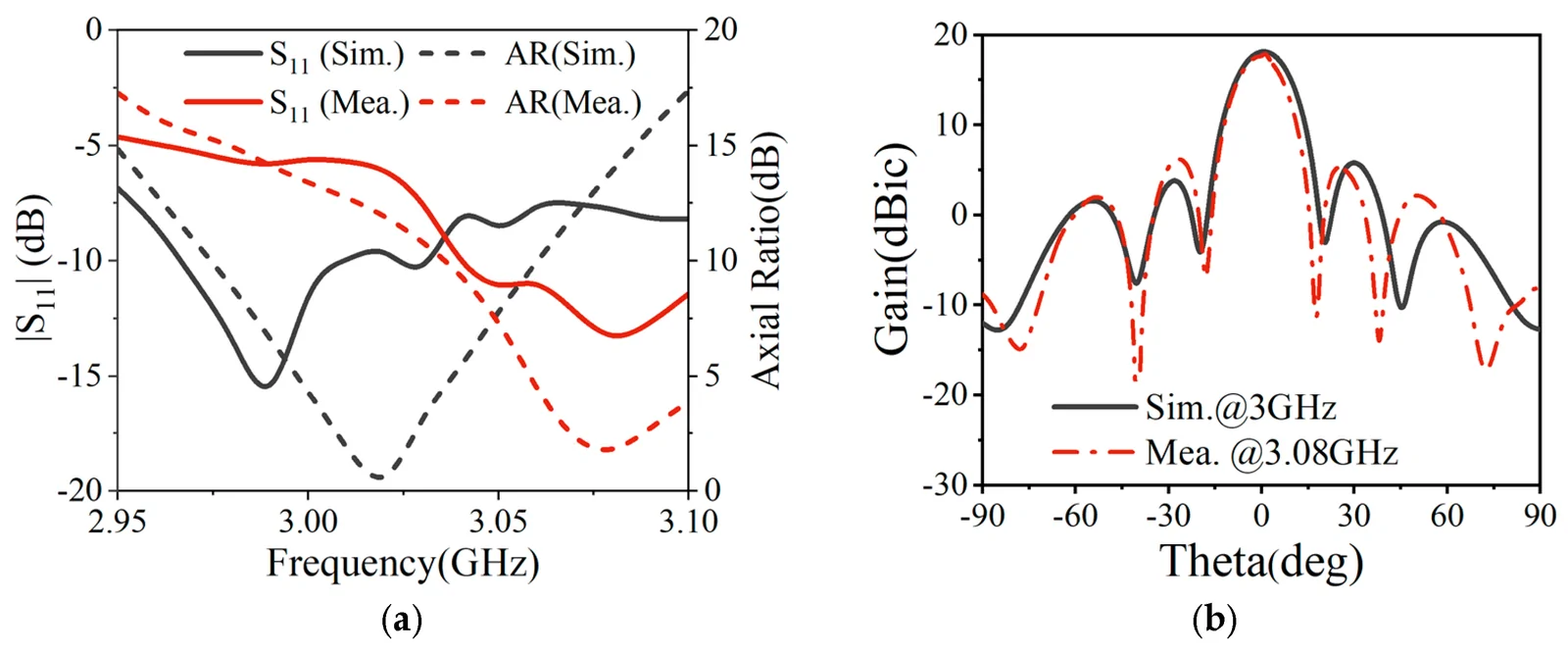
Simulated and measured (**a**) S-parameters and AR. (**b**) Gain at the resonate frequency.

**Figure 16:**
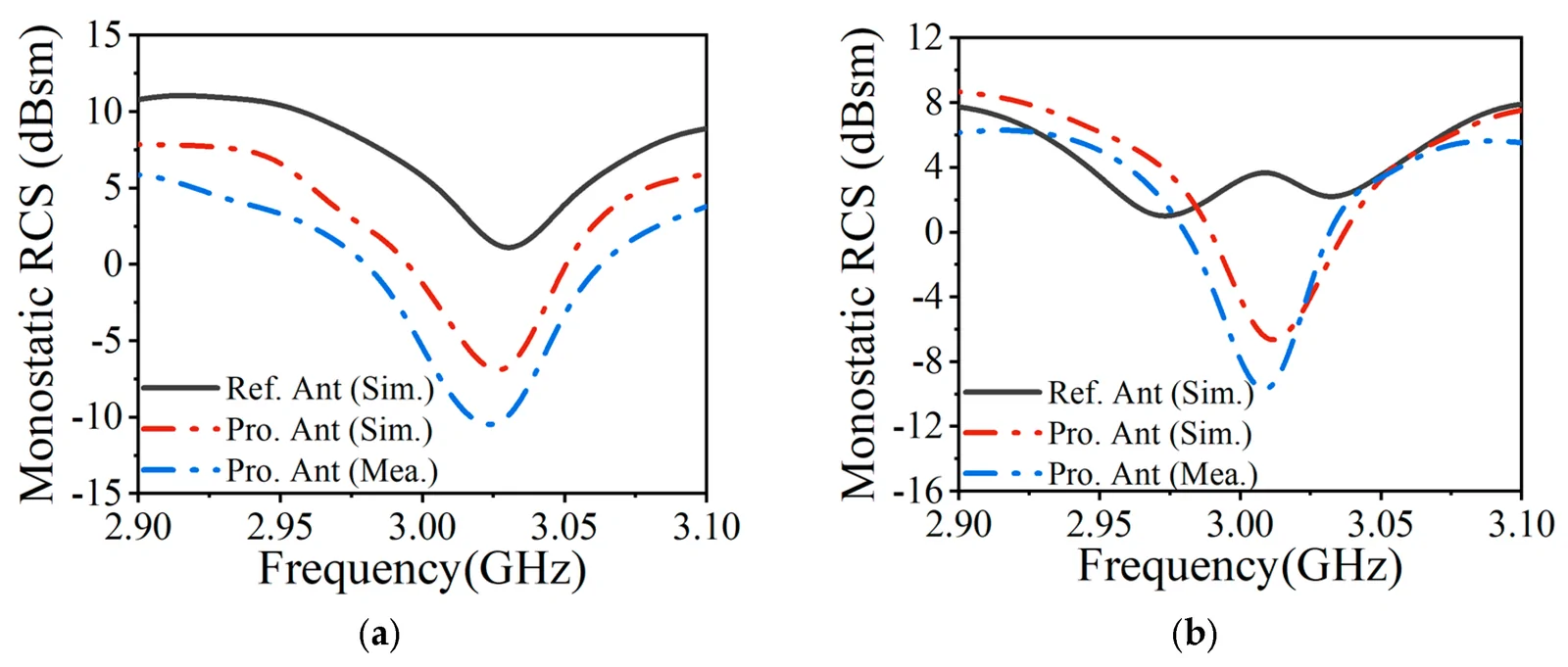
Simulated and measured monostatic RCS under normal incidences (**a**) *x*-polarization. (**b**) *y*-polarization.

**Figure 17:**
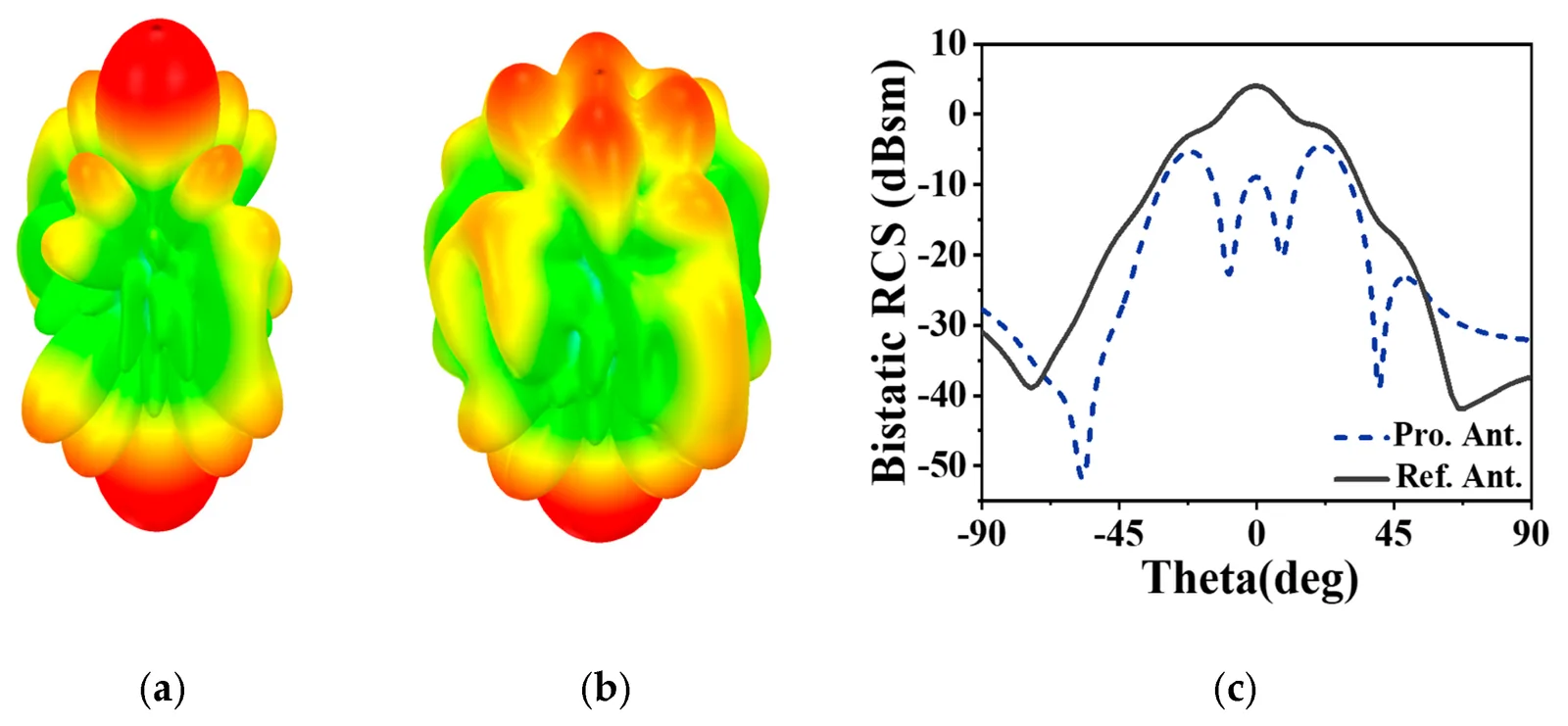
The bistatic RCS under y-polarized normal incidence. (**a**) 3-D pattern of reference array at 3 GHz. (**b**) 3-D pattern of proposed array at 3 GHz. (**c**) 2-D pattern of both arrays at 3 GHz.

**Table 1:** Parametric Sensitivity of the Parasitic Unit on the Reflection Phase under y-Polarized Normal Incidence at 3 GHz.

Parameter	Parametric Range (mm)	Reflected Phase Span	Sensitivity Level
*py*	31–32	+115° to −176°	High
*px*	30–31	−100° to −145°	Low
*ly*	3.5–3.6	−96° to −172°	High
*lx*	2.7–2.8	−150° to −171°	Low

**Table 2:** Comparison of the Previous Works.

Ref.	$f_{center}$	BW&AR Bandwidth	Profile ($\lambda_{0}$)	Aperture Efficiency	RCS Reference	RCS Reduction Bandwidth	Peak RCS Reduction	Method
[1]	6 GHz	3.5%	0.2	53.7%	Metal plate	96% (>10 dB)	20 dB	Loading resistors
[8]	5.5 GHz	50%	0.07	43.9%	Metal plate	111% (>5 dB)	24 dB	MTS-inspired
[11]	4.5 GHz	2.2%	0.1	39.3%	Metal plate	68% (>10 dB)	25 dB	PCM-inspired
[12]	9 GHz	94%	0.1	45.7%	Metal plate	78% (>10 dB)	/	PCM-inspired
[15]	9 GHz	20.1%	0.2	29.2%	Metal plate	139% (>0 dB)	/	Loading PCM
This work	3 GHz	1.5%	0.03	58.1%	Antenna	1.5% (>6 dB)	12.8 dB	CP antenna as MTS

## Data Availability

The original contributions presented in this study are included in the article. Further inquiries can be directed to the corresponding author.

## Abbreviations

The following abbreviations are used in this manuscript:

CP	Circularly polarized
RCS	Radar cross section
LEO	Low earth orbit
UAV	Unmanned aerial vehicles
PCM	Polarization conversion metasurfaces
AMC	Artificial magnetic conductors
LP	Linearly polarized
MS	Modal significance
CA	Characteristic angle
MTS	Metasurface
